# Does Tumor Size Influence the Diagnostic Accuracy of Ultrasound-Guided Fine-Needle Aspiration Cytology for Thyroid Nodules?

**DOI:** 10.1155/2016/3803647

**Published:** 2016-09-27

**Authors:** Do Hoon Koo, KwangSeop Song, Hyungju Kwon, Dong Sik Bae, Ji-hoon Kim, Hye Sook Min, Kyu Eun Lee, Yeo-Kyu Youn

**Affiliations:** ^1^Department of Surgery, Seoul National University Boramae Medical Center, 20 Boramae-ro 5-gil, Dongjak-gu, Seoul 156-70, Republic of Korea; ^2^Department of Surgery, Haeundae Paik Hospital, Inje University College of Medicine, 875 Haeundae-ro, Haeundae-gu, Busan 612-030, Republic of Korea; ^3^Department of Surgery, Seoul National University Hospital, College of Medicine, 101 Daehak-ro, Jongno-gu, Seoul 110-744, Republic of Korea; ^4^Cancer Research Institute, Seoul National University, College of Medicine, 101 Daehak-ro, Jongno-gu, Seoul 110-744, Republic of Korea; ^5^Division of Surgery, Thyroid Center, Seoul National University Cancer Hospital, 101 Daehak-ro, Jongno-gu, Seoul 110-744, Republic of Korea; ^6^Department of Radiology, Seoul National University Hospital, College of Medicine, 101 Daehak-ro, Jongno-gu, Seoul 110-744, Republic of Korea; ^7^Department of Pathology, Seoul National University Hospital, College of Medicine, 101 Daehak-ro, Jongno-gu, Seoul 110-744, Republic of Korea

## Abstract

*Background*. Fine-needle aspiration cytology (FNAC) is diagnostic standard for thyroid nodules. However, the influence of size on FNAC accuracy remains unclear especially in too small or too large thyroid nodules. The objective of this retrospective cohort study was to investigate the effect of nodule size on FNAC accuracy.* Methods. *All consecutive patients who underwent thyroidectomy for nodules in 2010 were enrolled. FNAC results (according to the Bethesda system) were compared to pathological diagnosis. The nodules were categorized into groups A–E on the basis of maximal diameter on ultrasound (≤0.5, >0.5–1, >1-2, >2–4, and >4 cm, resp.).* Results.* There were 502 cases with 690 nodules. Overall FNAC sensitivity, specificity, positive predictive value, negative predictive value, and accuracy were 95.4%, 98.2%, 99.4%, 86.4%, and 96.0%, respectively. False-negative rates (FNRs) of groups A–E were 3.2%, 5.1%, 1.3%, 13.3%, and 50%, respectively. Accuracy rates of groups A–E were 96.8%, 94.8%, 99%, 94.7%, and 87.5%, respectively.* Conclusion.* Although accuracy rates of FNAC in thyroid nodules smaller than 0.5 cm are comparable to the other group, thyroid nodules larger than 4 cm with benign cytology carry a higher risk of malignancy, which suggest that those should be considered for intensive follow-up or repeated biopsy.

## 1. Introduction

Fine-needle aspiration cytology (FNAC) is a first-line diagnostic test and cost-effective tool for the differential diagnosis of thyroid nodules [1–3]. FNAC was introduced in the 1950s [4] and has become a commonly used procedure, especially after the development of ultrasound- (US-) guided FNAC, which further improved the diagnostic accuracy of FNAC [5, 6]. US particularly facilitates accurate FNAC on nonpalpable small (≤1 cm) thyroid nodules. This has greatly improved the diagnosis and surgical treatment of papillary thyroid microcarcinoma (PTMC) [7–9].

However, the diagnostic accuracy of FNAC for large (>3-4 cm) and very small (i.e., subcentimeter) nodules remains unclear. Several studies report that the diagnostic accuracy of FNAC in easily targeted large thyroid nodules is significantly lower than it is in smaller nodules [10–12]. In fact, Pinchot et al. argued that all thyroid nodules that exceed 4 cm should be surgically removed due to the high false-negative FNAC rates that associate with such large thyroid nodules [11]. However, Shrestha et al. reported that >4 cm thyroid nodules did not associate with an increased risk of false-negative FNA results or a higher overall risk of malignancy [13]. With regard to very small nodules, Mazzaferri and Sipos proposed that FNAC should not be performed on 5 mm thyroid nodules because of the high rate of nondiagnostic FNAC [14]. However, Shrestha et al. did not find that small thyroid nodule size significantly influenced the risk of false-negative FNAC results, although they did observe a trend toward a higher false-negative rate (FNR) in the subcentimeter nodules [13].

The objective of this retrospective cohort study was to investigate the diagnostic accuracy of US-guided FNAC for thyroid nodules that were classified into several size categories (≤0.5, >0.5–1, >1-2, >2–4, and >4 cm, resp.).

## 2. Methods

### 2.1. Patient Selection

To determine the study cohort, all consecutive patients who underwent thyroidectomy for thyroid nodules between January 2010 and December 2010 in SNUH were identified. Patient gender, age, nodule location, and nodule size (as determined by US or, in one case, computed tomography because the nodule was so large), the FNAC results, and the final pathology results were collected retrospectively from the electronic medical records.

### 2.2. Inclusion and Exclusion Criteria

The FNAC was performed either by the radiologist in SNUH or in another hospital in the case of referred patients. Our institution's FNAC guidelines are patients with (1) >5 mm nodules with high-risk history, (2) abnormal cervical lymph nodes, (3) ≥1 cm nodules with microcalcifications present in nodule, (3) ≥1 cm nodules with solid and hypo-, iso-, or hyperechoic nodule, (4) ≥1.5 cm nodules with mixed cystic-solid nodule with any suspicious ultrasound features, and (5) ≥2 cm nodules with spongiform nodule. Thyroid nodule FNA cytology was reported using diagnostic groups outlined in the Bethesda System for Reporting Thyroid Cytopathology. All thyroid nodules that were included in the study were thoroughly reevaluated for FNAC results and radiological and pathological outcomes. Only the referred cases whose cytological slide was reviewed by the pathologist in our hospital were included in the study cohort. Patients were excluded if their FNAC report did not sufficiently describe the location of the thyroid nodules, their thyroidectomy was for a large symptomatic goiter without FNAC, the thyroidectomy was a completion thyroidectomy, or the surgery was for recurrence. The surgical indication for those nodules was based on Bethesda classification, for category 6 (consistent with malignant), 5 (suspicious malignancy), 4 (suspicious of follicular or Hürthle cell neoplasm), two consecutive series of 3 (atypia of undetermined significance) with suspicious sonographic feature and 1 (nondiagnostic) with solid, or 2 (benign) with symptomatic goiter or patients who were concerned about cosmetic problem.

### 2.3. Study Protocol

The enrolled thyroid nodules were classified into five groups according to their maximal diameter (*d*) on US (or computed tomography in one case): in groups A, B, C, D, and E, *d* was ≤0.5, >0.5–1, >1-2, >2–4, and >4 cm, respectively. To assess the accuracy of the FNAC, nodules whose FNAC results were benign, suspicious of malignancy, and malignant were included; nodules whose FNAC results were nondiagnostic, atypia of undetermined significance (AUS), or suspicious follicular neoplasm (FN) were excluded for calculating the accuracy. The nodules were also categorized as benign or malignant on the basis of the pathological results. The benign category included nodular hyperplasia, thyroiditis, or follicular adenoma. The malignant category included papillary thyroid carcinoma (PTC), follicular thyroid carcinoma (FTC), medullary thyroid carcinoma (MTC), or anaplastic thyroid carcinoma (ATC). This retrospective cohort study was approved by the Institutional Review Board of the Biomedical Research Institute of Seoul National University Hospital (SNUH). The requirement for informed consent was waived because of the retrospective nature of the study. The study was conducted in accordance with the ethical principles of the 1975 Declaration of Helsinki of 1975 as revised in 1983.

### 2.4. Statistical Analysis

All statistical evaluations were performed using the SPSS software package. Fisher's exact test was used to detect differences between groups in terms of sensitivity, specificity, positive predictive value (PPV), and negative predictive value (NPV). The Cochran-Armitage test was used to examine associations between sensitivity, specificity, PPV, and NPV and size. *p* values of <0.5 were defined as statistically significant.

## 3. Results

**Figure 1:**
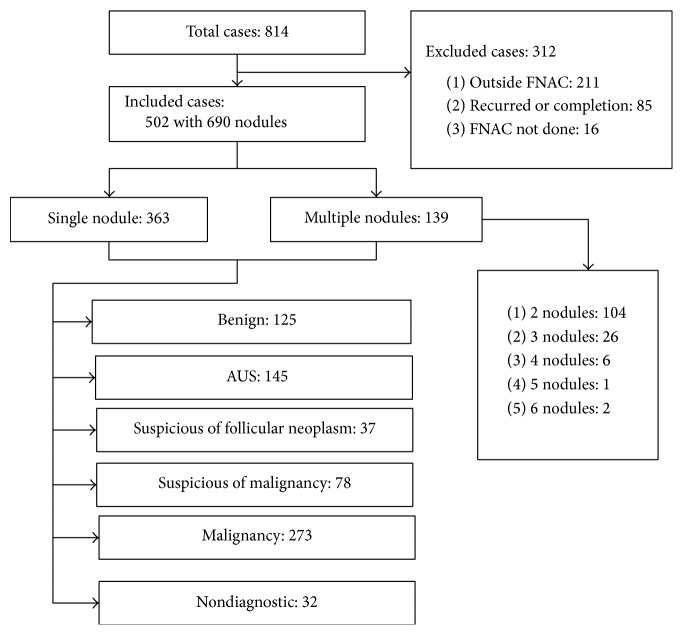
The patients and thyroid nodules in the present study. FNAC: fine-needle aspiration cytology; AUS: atypia of undetermined significance.

In total, 814 patients underwent thyroidectomy during the study period. Of these, 211 were excluded because their FNAC had been performed in another hospital and the slide was not reviewed in our hospital or their FNAC report did not sufficiently describe the location of the thyroid nodule(s). Sixteen patients who underwent thyroidectomy for a large symptomatic goiter without FNAC were also excluded. In addition, 40 cases of completion thyroidectomy and 45 cases of operation for recurrence were excluded. Thus, the study cohort consisted of 502 patients. In 363 and 139 patients, the FNAC was performed on single and multiple thyroid nodules, respectively. The latter consisted of 104 cases with two nodules, 26 cases with three nodules, six cases with four nodules, one case with five nodules, and six cases with six thyroid nodules. Overall, 690 thyroid nodules were included in this study (Figure 1).

### 3.1. Correlation between Cytological and Pathological Results

The 502 patients were on average 48.4 (range, 11–77) years old, and there were 424 females (84.5%). The mean nodule size was 1.3 cm (range, 0.2–9.4 cm). The pathological classification of all thyroid nodules according to FNAC category is shown in Table 1. The nodules were also classified into six categories on the basis of the FNAC results (which were reported according to the Bethesda system) as follows: there were 32 (4.6%) nondiagnostic nodules, 125 (18.1%) benign nodules, 145 (21.0%) AUS nodules, 37 (5.4%) suspicious FN nodules, 78 (11.3%) suspicious of malignancy nodules, and 273 (39.6%) malignant nodules. The malignancy rates for the nondiagnostic, benign, AUS, suspicious FN, suspicious malignancy, and malignant nodules were 53% (17/32), 13.6% (17/125), 55% (80/145), 32% (12/37), 97% (76/78), and 100% (273/273), respectively. The numbers of benign and malignant thyroid nodules in each size-FNAC category are shown in Table 2. The 690 nodules were divided into size categories A–E as follows: there were 145 (21.0%) group A nodules, 268 (38.8%) group B nodules, 169 (24.5%) group C nodules, 80 (11.6%) group D nodules, and 28 (4.1%) group E nodules.

### 3.2. Diagnostic Accuracy according to Thyroid Nodule Size

The diagnostic indices of FNAC in the different thyroid nodule size categories are shown in Table 3. In group A, the sensitivity, specificity, PPV, NPV, and diagnostic accuracy were 96.8%, 100%, 100%, 76.9%, and 96.8%, respectively. In group B, these values were 94.9%, 93.9%, 98.8%, 77.5%, and 94.8%, respectively. In group C, these values were 98.7%, 100%, 100%, 97.0%, and 99.0%, respectively. In group D, these values were 86.7%, 100%, 100%, 92%, and 94.7%, respectively. In group E, these values were 50%, 100%, 100%, 85.7%, and 87.5%, respectively. Therefore, overall FNAC sensitivity, specificity, positive predictive value, negative predictive value, and accuracy were 95.4%, 98.2%, 99.4%, 86.4%, and 96.0%, respectively. In groups A–E, the FNR was 3.2%, 5.1%, 1.3%, 13.3%, and 50%, respectively. Thus, sensitivity declined as thyroid nodule size increased despite a slight increase in group C. The sensitivity was especially low in group E (50%), and group E had a significantly lower sensitivity than the other size-based groups (*p* = 0.006). Group B had the lowest specificity and PPV (93.3% and 98.8%, resp.) of all groups. In the other four groups, the specificity and PPV were both 100%. However, these differences between group B and the other groups did not achieve statistical significance (*p* = 0.575 for specificity and *p* = 0.745 for PPV). Groups A and B had the lowest NPV (76.9% and 77.5%, resp.). The NPV in the other groups ranged from 85.7% to 97%. However, these differences were not significant (*p* = 0.076).

### 3.3. Comparison of FNAC Sensitivity

The Cochran-Armitage test showed that FNAC sensitivity tended to decline as the maximal diameter of thyroid nodule increased (*p* = 0.042). However, such tendencies were not observed for specificity and PPV (*p* = 0.238 and 0.945, resp.). To compare groups A and B in terms of diagnostic accuracy, we performed a subgroup analysis. However, the two groups did not differ significantly in terms of sensitivity, specificity, PPV, or NPV (*p* = 0.344, 0.585, 0.420, and 0.619, resp.).

## 4. Discussion

The influence of nodule size on the diagnostic accuracy of FNAC for thyroid nodules was unclear especially in too small or too large thyroid nodules. In terms of larger tumors, several studies report that even when the thyroid nodules are not difficult to target, the diagnostic accuracy of FNAC in >3-4 cm nodules is significantly lower than the accuracy in smaller nodules [10–12]. One of these studies was by McCoy et al., who were the first to study false-negative FNAC rates of larger nodules [12]. They found that these rates were markedly higher in ≥4 cm nodules than in smaller nodules. Meanwhile, Yoon et al. mentioned that false-negative FNACs were prevalent in larger nodules (>3 cm) because large nodules harbored eccentric malignant foci of PTC that were often not sampled [15]. By contrast, Shrestha et al. reported that thyroid nodule size greater than 4 cm did not increase the risk of false-negative FNAC results or the overall risk of malignancy [13]. Bohacek et al. also did not observe that larger nodule size tended to increase the false-negative FNA rate [16]. However, our study showed that the FNRs in the largest nodules (groups D and E) were considerable (13.3% in group D and 50% in group E): the overall FNR for nodules exceeding 2 cm was 21.1%. The very high FNR of FNAC for >4 cm thyroid nodules suggests that larger nodules may warrant further intensive follow-up or repeated biopsy.

We also observed that FNAC was significantly less sensitive in >4 cm thyroid nodules (*p* = 0.006). Furthermore, from Cochran-Armitage test, FNAC sensitivity also tended to decline as the nodule size increased (*p* = 0.042). Thus, although the number of the larger nodules in this study was relatively small, our observations are consistent with those of McCoy et al. and others [10–12], namely, that nodule size does affect FNAC accuracy. This suggests that larger thyroid nodules (>4 cm) may require diagnostic thyroid lobectomy.

The marked increase in the incidence of PTMC recently has led to concerns about the diagnosis and treatment of these small nodules. The American thyroid association guidelines do not recommend routine FNAC for subcentimeter nodules. This is largely due to the low risk of malignancy in smaller lesions and the unacceptably high rate of nondiagnostic FNAC in such small nodules [14, 17]. Our institution's guidelines are similar: we perform FNAC on >5 mm thyroid nodules in high-risk patients or the sonographically suspicious nodule. In our study, a relatively large proportion of the sampled nodules were of subcentimeter size. Specifically, 21% of all nodules (145/690) were less than 5 mm. This high rate may largely reflect tumor multiplicity, size reduction for postoperative measurement, requests by the patient to diagnose a suspicious nodule, and the relatively high rate of referred cases as tertiary centers.

The diagnostic accuracy of FNAC for thyroid nodules that are less than 1 cm in diameter is reported to be lower than for those with diameter exceeding 1 cm [5, 13, 18]. Nevertheless, Kim et al. and several other authors showed that FNAC is still a useful tool for subcentimeter nodules [19–21]. In our study, approximately 60% (413/690) of the nodules were subcentimeter nodules. When we analyzed the diagnostic indices of FNAC in this large cohort, we found that the nondiagnostic rate of FNAC was low for all nodules (4.6%, 32/690) and that the subcentimeter nodules did not differ significantly from larger nodules in terms of FNAC specificity, PPV, NPV, and diagnostic accuracy. Moreover, when we compared groups A (<0.5 cm) and B (>0.5–1 cm) in terms of the rate of diagnostic accuracy, we did not find significant differences in sensitivity, specificity, PPV, or NPV (*p* = 0.344, 0.585, 0.420, and 0.619, resp.). This suggests that FNAC for subcentimeter nodules is sufficiently technically feasible to be used to predict thyroid malignancy. However, the FNR in these small nodules was still considerable (3.2% for group A and 5.1% for group B). This may largely reflect difficulties targeting the nodule or discordance between the US features and the pathology results. In addition, clinically suspicious subcentimeter nodules were more submitted to FNAC that may represent selection bias. It could be another explanation that our result showed relatively lower level of FNR for those nodules.

In all of the cases in the present study, both the radiological and pathological diameters were measured and compared. For this, a radiologist and a pathologist from our institution, who were coauthors of this article, carefully compared the US images with the pathology slides. There was good agreement between locations of the tumor site on the preoperative FNAC and postoperative pathology report in all cases. However, the US diameter was used in the analyses of the present study because US-guided FNAC is performed in clinical practice to help make treatment decisions about suspicious thyroid nodules [11, 22]. Meanwhile, the pathological diameter of the predominantly cystic nodule can change after tumor resection, thus affecting the results on the pathological slide.

The risk of malignancy in the Bethesda FNAC categories in our patients was 13.6% for benign nodules, 50% for AUS nodules, and 98% for suspicious malignancy nodules. These risks were relatively higher than those noted in other reports [16, 23, 24]. In the 17 cases in which FNAC was benign but the final pathological outcome was malignancy, the risk of malignancy for papillary carcinoma was 10.4% excluding three cases of FTC and one case of MTC, which was not available or difficult to diagnose preoperatively. This may reflect the fact that our patients were treated in a tertiary referral center and/or the conservative tendency of pathologists in terms of deciding the FNAC result.

Actually from this study, only the cases which underwent thyroidectomy were included; in other words, follow-up cases for benign or atypical FNAC results were not included. Eventually, the subjects with small nodules which were benign cytology did not undergo surgery. Therefore, false-negative results were not detected from those cases, which might be considered as our study limitation. Even if those nodules were included for the study, FNR might be slightly increasing approximately 5% in overall sized thyroid nodules. In this study, we aimed to investigate all consecutive thyroidectomized patients in our institution and prove the diagnostic accuracy of FNAC for those nodules and achieved comparable outcomes to previous reports. As shown in the methods, nodules smaller than 1 cm with benign feature on US were not an indication of FNAC or a candidate for surgery either. Therefore, it was neither reasonable nor suitable for this study to investigate FNR of all thyroid nodules which was not to be a surgical candidate.

This study has other several limitations. First, study design was not prospective. Second, several nodules that had undergone FNAC outside our hospital were included even though the result had been subsequently reviewed in our hospital. Third, in cases where FNAC was performed on multiple thyroid nodules, the radiopathological correlation for each individual nodule was complex even though both a radiologist and a pathologist reviewed the US images and pathological slides thoroughly.

## 5. Conclusions

Although the accuracy rates of FNAC in thyroid nodules smaller than 0.5 cm are comparable to the other size of thyroid nodules, thyroid nodules larger than 4 cm with benign cytology carry a higher risk of malignancy. Therefore, the patient with thyroid nodules with >4 cm and benign cytology should be considered for intensive follow-up or repeated biopsy after consideration of clinical and sonographic features.

## Figures and Tables

**Table 1:** Final pathological results of the nodules divided according to the Bethesda fine-needle aspiration cytology (FNAC) categories.

	Nondiagnostic (*n* = 32)	Benign (*n* = 125)	AUS (*n* = 145)	Suspicious FN (*n* = 37)	Suspicious malignancy (*n* = 78)	Malignancy (*n* = 273)

NH (*n* = 182)	12	101	55	13	1	0
Thyroiditis (*n* = 16)	3	6	5	2	0	0
FA/HA (*n* = 17)	0	1	5	10	1	0
FTC (*n* = 25)	0	3	10	9	1	2
MTC (*n* = 9)	0	1	6	0	1	1
PTC (*n* = 440)	17	13	64	3	73	270
ATC (*n* = 1)	0	0	0	0	1	0

Malignant risk (%)	53	13.6	55	32	97	100

NH: nodular hyperplasia; FA: follicular adenoma; HA: Hürthle cell adenoma; FTC: follicular thyroid carcinoma; MTC: medullary thyroid carcinoma; PTC: papillary thyroid carcinoma; ATC: anaplastic thyroid carcinoma; AUS: atypia of undetermined significance.

**Table 2:** Number of benign and malignant thyroid nodules in the different size and Bethesda reporting system categories.

Max. diameter	Nondiagnostic (*n* = 32)	Benign (*n* = 125)	AUS (*n* = 145)	Suspicious FN (*n* = 37)	Suspicious malignancy (*n* = 78)	Malignancy (*n* = 273)
	Benign/malignant	Benign/malignant	Benign/malignant	Benign/malignant	Benign/malignant	Benign/malignant
≤0.5 cm (*n* = 145)	4/9	10/3	3/23	1/0	0/25	0/67
>0.5–1 cm (*n* = 268)	6/5	31/9	11/31	3/2	2/36	0/132
>1-2 cm (*n* = 169)	3/3	32/1	26/16	12/2	0/10	0/64
>2–4 cm (*n* = 80)	1/0	23/2	19/8	7/7	0/4	0/9
>4 cm (*n* = 28)	1/0	12/2	6/2	2/1	0/1	0/1

AUS: atypia of undetermined significance; *d*: diameter; FN: follicular neoplasm.

The maximal diameter of the nodule on preoperative ultrasound was used for this analysis.

**Table 3:** Diagnostic indices of fine-needle aspiration cytology (FNAC) in the five thyroid nodule size categories.

Group	A (≤0.5 cm)	B (>0.5–1 cm)	C (>1-2 cm)	D (>2–4 cm)	E (>4 cm)	*p* value
Sensitivity	96.8	94.9	98.7	86.7	50	0.006
Specificity	100	93.9	100	100	100	0.575
PPV	100	98.8	100	100	100	0.745
NPV	76.9	77.5	97	92	85.7	0.076
Diagnostic accuracy	96.8	94.8	99	94.7	87.5	—

PPV: positive predictive value; NPV: negative predictive value.
